# An Electrochemical Sensor of Poly(EDOT-pyridine-EDOT)/Graphitic Carbon Nitride Composite for Simultaneous Detection of Cd^2+^ and Pb^2+^

**DOI:** 10.3390/ma11050702

**Published:** 2018-04-29

**Authors:** Shuai Ding, Ahmat Ali, Ruxangul Jamal, Ling Xiang, Ziping Zhong, Tursun Abdiryim

**Affiliations:** 1 Key Laboratory of Petroleum and Gas Fine Chemicals, Educational Ministry of China, College of Chemistry and Chemical Engineering, Xinjiang University, Urumqi 830046, China; dingshuai910526@sina.com (S.D.); ahmatjanchem@126.com (A.A.); xiangling8023@sina.com (L.X.); zzp1037934439@sina.com (Z.Z.); 2 Key Laboratory of Functional Polymers, Xinjiang University, Urumqi 830046, China

**Keywords:** poly(BPE)/g-C_3_N_4_ composite, DPV, electrochemical sensor, simultaneous detection, heavy metals

## Abstract

In this study, poly(2,5-bis(3,4-ethylenedioxythienyl)pyridine)/graphitic carbon nitride composites (poly(BPE)/g-C_3_N_4_) were prepared by an in situ chemical polymerization method. Composites were characterized by using Fourier transform infrared spectroscopy (FT-IR), ultraviolet–visible absorption spectra (UV–vis), X-ray diffraction (XRD), energy-dispersive X-ray spectroscopy (EDS), scanning electron microscopy (SEM), and transmission electron microscopy (TEM). Furthermore, electrochemical sensors were applied for the electrochemical determination of Cd^2+^ and Pb^2+^ using the differential pulse voltammetry (DPV) method. The results indicated that 10 wt % poly(BPE)/g-C_3_N_4_ composite-modified electrode exhibited linear detection ranging from 0.12 to 7.2 μM and 0.08 to 7.2 μM for Cd^2+^ and Pb^2+^, with detection limits (S/N = 3) of 0.018 μM and 0.00324 μM. Interference analysis suggested that the 10 wt % poly(BPE)/g-C_3_N_4_-modified electrode can be applied for the detection of the Cd^2+^ and Pb^2+^ in real samples.

## 1. Introduction

Industrial waste water usually contains heavy metal ion—such as Zn^2+^, Cd^2+^, Pb^2+^, Cu^2+^ and Hg^2+^, the accumulation of these heavy metal ions in the human body can cause many chronic diseases [1,2,3]. For example, an imbalance of Zn^2+^ can reduce the amount of vitamin C and iron in the body, and cause iron deficiency anemia. Cd^2+^ accumulation to a certain value in the human body will lead to renal failure. Both lead and mercury affect the brain and nervous system, and result in mental retardation and brain damage in children [4]. Therefore, developing a simple and sensitive analytical method to detect these heavy metal ions is essential [5].

Up to now, various techniques have been used for detection of heavy metals, including inductively coupled plasma mass spectrometry [6], inductively coupled plasma atomic emission spectrometry [7], atomic fluorescence spectrometry [8], atomic absorption spectrometry [9], surface enhanced Raman spectrometry, and electrochemical analysis technology [5,10]. However, most of these methods require expensive equipment, complicated procedures, and specialized training. Due to its high sensitivity, low operating cost, and fast response, electrochemical analysis has been considered as an effective technique for the determination of heavy metal ions. Among the current developed electrochemical approaches, anodic stripping voltammetry (ASV) has been widely accepted as one of the most simple and efficient techniques, since it can be operated easily and can analyze several heavy metal ions simultaneously. The ASV includes two steps: the preconcentration step and the stripping step. During the preconcentration step, a low potential is applied, causing the metal ions to become concentrated on the working electrode and then reduced to their reduction states. During the stripping step, potential is changed from low potential to high potential, causing the reduced metal ions to change to their oxidation states, and the reduced metals are stripped from the electrode surface into the solution in their ionic forms. From the stripping peak potential and current, the metal ions can be qualitatively and quantitatively determined.

The sensitivity of the electrochemical sensor mainly relies on its electrochemical properties and the adsorption ability of ions. To improve the sensitivity of the electrode, novel sensing materials must be developed [5]. The ineffectiveness of conventional electrodes can be improved by modifying them with electrochemically active materials, such as metals [11], metal oxides [12], carbon materials [13], and conducting polymers [14]. Among them, carbon materials and their composites have great applications in the field of sensors [15,16,17]. In recent years, graphitic carbon nitride (g-C_3_N_4_) has drawn much attention for its simple preparation, good chemical stability, high catalytic activity, and green environmental protection material [18]. Besides that, g-C_3_N_4_ shows a two-dimensional structure constructed from tri-s-triazine units connected by planar amino groups, and it can be considered as containing nitrogen-substituted graphite hexatomic ring units [19]. Moreover, it has a structure similar to graphene, with layers connected to each other through weak van der Waals forces between the C–N covalent bonds. Because of its exceptional optical, thermal, electrical, mechanical, and chemically inert properties, applications of g-C_3_N_4_ are typically lithium-ion batteries [20], water splitting [21], fuel cells [22], photocatalysis [23], and electrochemiluminescence and fluorescent sensor fields [24,25].

Recently, more and more attention has been focused on the application of g-C_3_N_4_ in the field of electrochemical sensors [26,27,28]. It has been reported that metal ions can be absorbed on a g-C_3_N_4_ matrix through electrostatic bonding or coordination with several N-atoms in the tri-s-triazine ring [29]. However, the poor conductivity of g-C_3_N_4_ strongly effects its use in practical applications. Therefore, the application of g-C_3_N_4_ in electrochemical sensing is still challenging. Studies show that the conductivity of the g-C_3_N_4_ can be enhanced by using some electroactive materials, such as graphene, metal oxides, and conjugated polymers [17,30,31]. These composite materials can enhance the sensitivity and selectivity of the electrodes. Among these electroactive materials, conjugated polymers (such as polyaniline, polypyrrole, polythiophene) are widely used as electroactive materials with their unique π-conjugation system, high conductivity, and fast electron properties. In recent years, the donor–acceptor–donor (D–A–D) type of conjugated polymer has been considered an ideal substance because its donor and acceptor units regularly alternate connections in each structural unit. In addition, the D–A–D type of conjugated polymer contains O, S, and N atoms, which can donate two unpaired electrons. Thus, the polymer can coordinate readily to positively charged heavy metal ions. Therefore, the composite of a D–A–D type conjugated polymers and g-C_3_N_4_ can be used as an excellent electrode modification material for the efficient detection of heavy metal ions by electrochemical sensor.

In this work, poly(2,5-bis(3,4-ethylenedioxythienyl)pyridine)/graphitic carbon nitride composites (poly(BPE)/g-C_3_N_4_) were prepared by an in situ chemical polymerization method and applied to for the simultaneous detection of Cd^2+^ and Pb^2+^. The combination of poly(BPE) and g-C_3_N_4_ not only improved the conduction pathway on the electrode surface, but also produced a strong conjugating effect on them, thus enhancing the adsorption of metal ions. The metal ions could coordinate with the lone-pair electrons of nitrogen in the tri-s-triazine unit, and the nitrogen and sulfur atoms in poly(BPE) had similar effects. Overall, using this approach for the determination of Cd^2+^ and Pb^2+^ offers several advantages including low cost, simple preparation, high selectivity, good sensitivity, and reusability. This approach might be an alternative tool for heavy metal detection in environmental monitoring.

## 2. Materials and Methods

### 2.1. Materials

EDOT (3,4-ethylenedioxythiophene), n-butyl lithium, 3,6-dibromopyridazide, and ferric chloride were bought from Shanghai Chemical Reagents Company (Shanghai, China). All the other chemicals and solvents, including urea, zinc chloride, sodium acetate (NaAc), acetic acid (HAc), and chloroform were used as received without further purification. The 0.1 M acetate buffer solution (ABS) was obtain through mixing different volume ratio stock solutions of 0.1 M NaAc and HAc. Standard solutions of 1 × 10^−3^ M of Cd^2+^ and Pb^2+^ were prepared by dissolving lead acetate and cadmium acetate in ultrapure water, respectively.

### 2.2. Instruments and Characterizations

The structure and properties of the composites were investigated by Fourier transform infrared spectroscopy (FT-IR), ultraviolet–visible absorption spectra (UV–vis), X-ray diffraction (XRD), energy-dispersive X-ray spectroscopy (EDS), scanning electron microscopy (SEM), and transmission electron microscopy (TEM). The FT-IR spectra of the composites were recorded with a BRUKER-QEUINOX-55 FTIR spectrometer (Bruker, Billerica, MA, USA) using KBr pellets. The UV–vis spectra of the samples were recorded with a UV–vis spectrophotometer (UV4802, Unico, Dayton, NJ, USA). XRD patterns were obtained by using a Bruker AXS D8 diffractometer (Bruker, Billerica, MA, USA), scan range (2θ), which was 10–80° with monochromatic CuKα radiation source (λ = 0.15418 nm). The SEM images were carried out on a scanning electron microscope (SEM, Hitachi, Chiyoda-ku, Japan, S-4800, operating voltage, 5 kV), with powdered samples scattered on the conducting resin. Before SEM imaging, the samples were sputtered with thin layers of aurum under vacuum. Transmission electron microscopy (TEM, Akishima, Tokyo, Japan, model 2100) was performed with an accelerating voltage of 100 kV. The elemental percentages of samples were measured using energy-dispersive X-ray spectroscopy, which was taken on a Leo1430VP microscope (Carl Zeiss Inc., Oberkochen, Germany) with operating voltage 5 kV.

### 2.3. Preparation of g-C_3_N_4_ and Poly(BPE)/g-C_3_N_4_ Composites

#### 2.3.1. Preparation of g-C_3_N_4_

The graphitic carbon nitride samples were prepared by a pyrolysis method using urea as precursor [32]. Typically, 10 g of urea was loaded into a 40 mL crucible with a cover. The crucible was placed in a muffle furnace under air condition and heated to 500 °C with a heating rate of 10 °C/min. Then, it was heated at 500 °C for 2 h and at 550 °C for another 2 h. Finally, the crucible was naturally cooled to room temperature, and a pale-yellow powder was obtained. The sample was washed with deionized water and absolute ethanol three times and dried at 60 °C for 24 h.

#### 2.3.2. Preparation of Monomer BPE

**Scheme 1:**
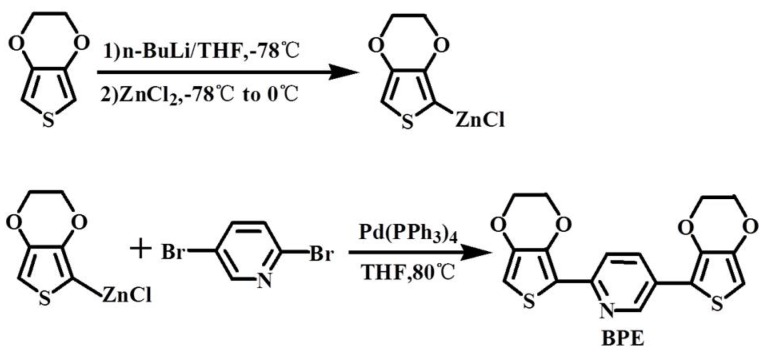
Synthesis route for the monomer of BPE.

**Figure 1:**
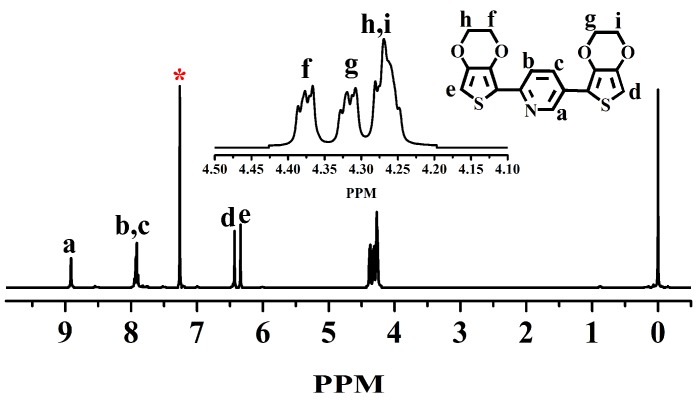
^1^H-NMR spectra of bis(3,4-ethylenedioxythienyl)pyridine (BPE) in CDCl_3_, starred peak come from CDCl_3_.

The monomer of BPE was synthesized based on the previous report and reaction process as shown in Scheme 1 [33]. EDOT (5.0 g, 35.2 mmol) was added to 100 mL of anhydrous THF. Upon cooling to −78 °C, n-BuLi was added to the solution drop-wise (14.7 mL, 2.4 M) and stirred for 60 min, then ZnCl_2_ (4.8 g, 16.7 mmol) was added. The mixture solution was stirred at 0 °C for 60 min before Pd(PPh_3_)_4_ (1.0 g, 0.865 mmol) and 2,5-dibromopyridine (2.08 g, 8.78 mmol) in 25 mL THF was added. The reaction mixture was stirred at 80 °C for another 72 h. Finally, the mixture was cooled to room temperature, and the THF was removed under reduced pressure. The product was extracted using chloroform, then further purified by recrystallization by methanol to obtain canary yellow solid. The structure of the monomer was characterized by ^1^H-NMR (in Figure 1). ^1^H-NMR: (400 MHz, CDCl_3_): δ [ppm] 4.30 (m, 8H), 6.33 (s, 1H), 6.43 (s, 1H), 7.91 (m, 2H), 8.91 (s, 1H).

#### 2.3.3. Preparation of Poly(BPE)/g-C_3_N_4_ Composites

To prepare the poly(BPE)/g-C_3_N_4_ composites, a certain weight of g-C_3_N_4_ was dispersed in 20 mL chloroform with ultrasonication for 30 min. Monomer BPE dissolved in 10 mL chloroform was added and ultrasonic dispersion applied for another 30 min. After that, FeCl_3_ was dispersed in 10 mL chloroform and added to the above solution drop by drop as oxidant. The reaction was stirred under magnetic stirring for 24 h. Finally, the sample was washed several times with chloroform, methanol, and distilled water, and then dried in an oven at 60 °C for 12 h.

Different weight percentages of poly(BPE)/g-C_3_N_4_ composite were obtained with a similar method. The pure poly(BPE) was prepared by a similar method without the addition of g-C_3_N_4_.

### 2.4. Preparation of Modified Electrodes

The bare glassy carbon electrode (GCE) was polished with 0.3 and 0.05 μm alumina slurries in sequence. The GCE was modified by a simple casting method. Five microliters of poly(BPE)/g-C_3_N_4_ dispersion (1 mg/mL) was spread onto the surface of the cleansed electrode and left to dry at 40 °C. The different weight percentages of poly(BPE)/g-C_3_N_4_, pure poly(BPE), and g-C_3_N_4_-modified GCE were prepared using the same procedure.

### 2.5. Electrochemical Measurements

All electrochemical measurements were carried out on CHI660C electrochemical workstation (ChenHua Instruments Co., Shanghai, China). In the three-electrode system, composite-modified electrode, platinum electrode, and saturated calomel electrode were used, respectively, as the working electrode, counter electrode, and reference electrode. Cyclic voltammograms (CV) were carried out in a mixing solution of 5 mM [Fe(CN)_6_]^3−/4−^ with 0.1 M KCl with a scanning rate of 50 mV·s^−1^. Differential pulse voltammetry (DPV) was performed in 0.1 M ABS (pH = 4.5) with potential interval −1.4 to −0.2 V; deposition potential, −1.4 V; deposition time, 210 s; pulse width, 50 ms; pulse period, 100 ms; increment potential, 2 mV.

## 3. Results and Discussion

### 3.1. Structure Characterization of Poly(BPE)/g-C_3_N_4_ Composites

**Figure 2:**
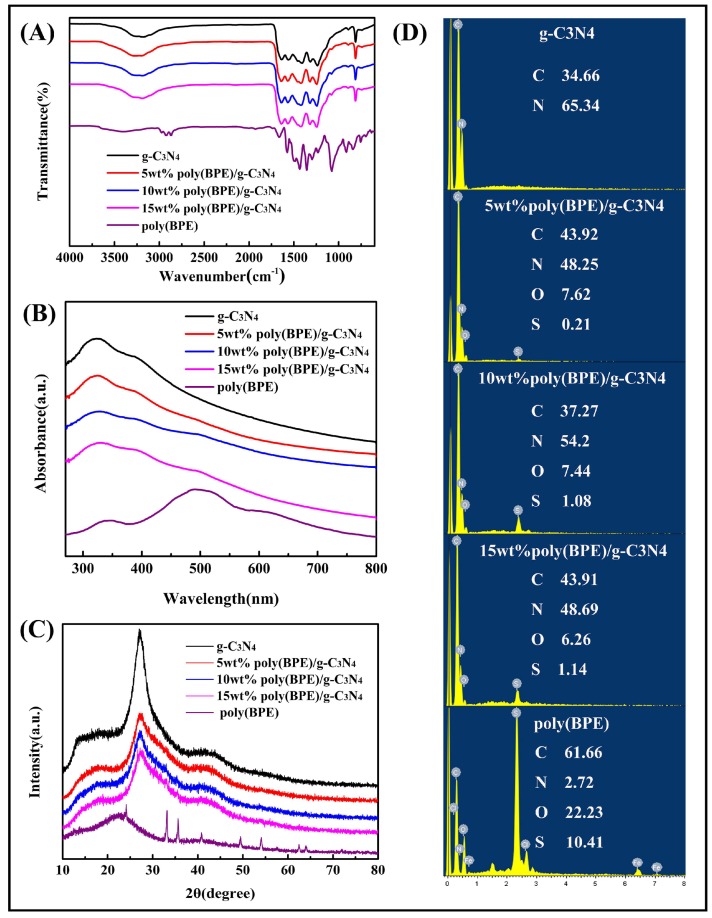
(**A**) FT-IR spectra; (**B**) UV–vis spectra; (**C**) XRD patterns; and (**D**) EDS of g-C_3_N_4_, using different weight percentages of poly(BPE)/g-C_3_N_4_ composites and poly(BPE).

Figure 2A represents the FT-IR spectra of g-C_3_N_4_, poly(BPE), and poly(BPE)/g-C_3_N_4_ composites. In the spectra of g-C_3_N_4_, the band at 812 cm^−1^ is attributed to the bending vibration of the triazine ring modes out of plane. The weak peak at 890 cm^−1^ is assigned to the cross-linked heptazine deformation mode [34]. The fingerprint regions at the range of 1230–1650 cm^−1^ correspond to the stretching vibrations of C–N and C=N in heterocycles [35]. A broad vibration band appears in the region of 3000–3500 cm^−1^, which can be assigned to the stretching vibrations of aromatic N–H bonds of the uncondensed amino group (–NH_2_) [36]. For the pure poly(BPE), the band at 2800–3100 cm^−1^ corresponds to the aromatic C–H stretching vibrations. The peaks at 1439, 1358, and 1300 cm^−1^ are due to the C=C asymmetric stretching vibration and C–C stretching vibration in the poly(BPE) ring. The bands at 1663 cm^−1^ and 1658 cm^−1^ are C=N stretching in the pyridine. There are only a few discrepancies between the spectra of g-C_3_N_4_ and poly(BPE)/g-C_3_N_4_ composite, and it is probably because the peaks of g-C_3_N_4_ and poly(BPE) at 1230–1650 cm^−1^ have been superimposed. The new weak band at 1081 cm^−1^ was observed after incorporating the polymer into the composite. This band is assigned to the presence of ν(C–O–C) in the ethylenedioxy group of EDOT, due to the strong interaction between poly(BPE) and g-C_3_N_4_ [37,38,39].

Figure 2B represents the UV–vis spectra of g-C_3_N_4_, poly(BPE), and poly(BPE)/g-C_3_N_4_ composites. The UV–vis spectra of g-C_3_N_4_ shows characteristic absorption peaks from 270 to 430 nm, which are characteristic peaks of the carbon nitride. Furthermore, the absorption peaks of poly(BPE) appear at 348, 490, and 586 nm, which are assigned to the π–π* transition of the thiophene ring [39]. In the case of poly(BPE)/g-C_3_N_4_ composites, aside from the characteristic peak of g-C_3_N_4_, the absorption peak appears at 490 nm for 10 wt % poly(BPE)/g-C_3_N_4_ and 15 wt % poly(BPE)/g-C_3_N_4_. The results suggested that poly(BPE) was successfully incorporated into the g-C_3_N_4_ matrix.

Figure 2C exhibits the XRD patterns of poly(BPE), g-C_3_N_4_, and poly(BPE)/g-C_3_N_4_ composites. The poly(BPE) shows broad characteristic peaks at about 2θ = 22.5°, which is assigned to the π–π* stacking within the molecule. Moreover, the sharp diffraction peaks at 2θ ≈ 33°, 35°, 41°, 49°, and 54° are associated with the FeCl_4_^–^ doping agent [38,39]. Diffraction peaks of g-C_3_N_4_ are located at 27.1° and 13.2°, which can be indexed as (002) and (100) diffraction planes for graphitic materials (JCPDS 87-1526). The strongest diffraction peak of graphite appears at 2θ = 27.1°, which is attributed to the planes of graphitic structures. The minor peak at 13° corresponds to the hole-to-hole arrays of tri-s-triazine units. For the composites, no other peaks appeared, indicating the crystal structure was not changed. However, it can be seen that as the intensity of (100) planes of g-C_3_N_4_ decreases, the amount of poly (BPE) increases, suggesting that the original ordered intralayer structures of g-C_3_N_4_ probably suffered from damage in the presence of poly(BPE) [40,41].

Figure 2D depicts the EDS of g-C_3_N_4_ and poly(BPE)/g-C_3_N_4_ composites and poly(BPE). As shown in Figure 2D, the EDS spectrum of the pure g-C_3_N_4_ samples reveal the existence of C and N elements [42]. In the spectrum of pure poly(BPE), the C, N, O, and S elements are the major chemical elements, and a small amount of Fe element was detected due to the addition of ferric chloride [38]. For the poly(BPE)/g-C_3_N_4_, except the C and N elements, the O and S elements were also detected, which provided powerful evidence for the existence of poly(BPE). In addition, as the percentage of poly(BPE) increases, the weight percentage of S element in the composite increases from 0.21% to 1.14%.

**Figure 3:**
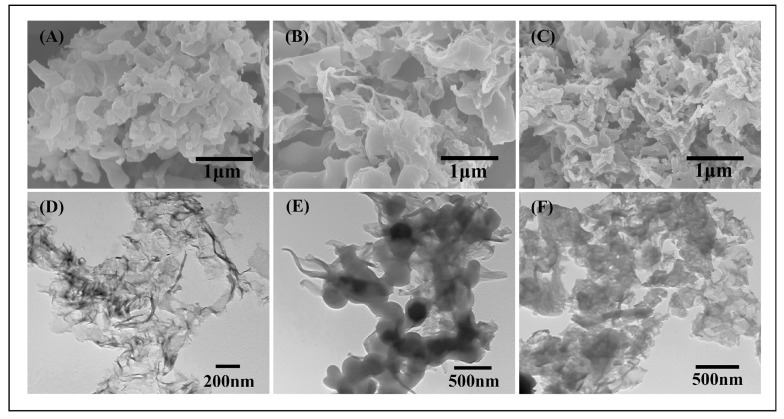
SEM images of (**A**) g-C_3_N_4_; (**B**) poly(BPE); (**C**) 10 wt % poly(BPE)/g-C_3_N_4_ composite; and TEM images of (**D**) g-C_3_N_4_; (**E**) poly(BPE); and (**F**) 10 wt % poly(BPE)/g-C_3_N_4_ composite.

Figure 3 presents the SEM and TEM images of g-C_3_N_4_, poly(BPE), and 10 wt % poly(BEP)/g-C_3_N_4_ composite. As shown in Figure 3A,D, the sheet-like g-C_3_N_4_ was like the fold of the sheet structure and appears as ultrathin and well-spread sheets. As shown in Figure 3B,E, the pure poly(BPE) exhibits uneven thickness of the irregular lump material. As shown in Figure 3C,F, slightly less poly(BPE)/g-C_3_N_4_ (at 10 wt %) grows on the surface of g-C_3_N_4_ compared to pure g-C_3_N_4_ and poly(BPE), forming a net-like structure. Due to the richness of amino, g-C_3_N_4_ can be easily dispersion by the method of ultrasonication. The BPE monomer can be adsorbed on the surface of sheet-like g-C_3_N_4_ by the π–π aromatic interaction and electrostatic attraction. During the in situ chemical polymerization from the effect of ferric chloride, the monomer adsorbed on the surface of g-C_3_N_4_ grew on g-C_3_N_4_ to form poly(BPE) [43].

### 3.2. Electrochemical Characterization of Poly(BPE)/g-C_3_N_4_ Composites

**Figure 4:**
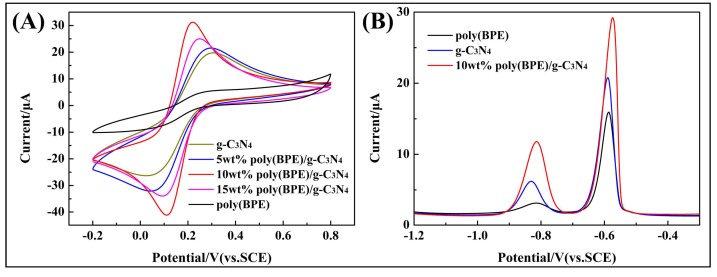
(**A**) Cyclic voltammograms measured with g-C_3_N_4_, poly(BPE), poly(BPE)/g-C_3_N_4_ composite-modified glassy carbon electrode (GCE) in a solution of 5 mM Fe(CN)_6_^3−/4−^ containing 0.1 M KCl. (**B**) Differential pulse voltammogram (DPV) of g-C_3_N_4_, poly(BPE), 10 wt % poly(BPE)/g-C_3_N_4_ composite-modified GCE in 0.1 M acetate buffer solution (ABS) (pH = 4.5) containing 2 μM of Cd^2+^ and Pb^2+^. Deposition potential: −1.4 V, deposition time: 210 s, pulse width: 50 ms; pulse period: 100 ms; increment potential: 2 mV.

The electrochemical activity of differently modified GCEs was evaluated using CV in a redox probe solution containing 5 mM [Fe(CN)_6_]^3−/4−^ in 0.1 M KCl. As shown in Figure 4A, all the modified electrodes show well-defined redox peaks, which are related to the [Fe(CN)_6_]^3−/4−^ redox processes. Compared to poly(BEP) and g-C_3_N_4_-modified GCE, the redox peak of the poly(BEP)/g-C_3_N_4_ composite-modified GCE is obviously enhanced. This indicates that more electrochemically active sites were present on the surface of poly(BEP)/g-C_3_N_4_. Also, it is probably due to the excellent electron transfer property of poly(BEP) [44]. The peak-to-peak potential (∆E_p_ = E_anodicpeak_ − E_cathodicpeak_) at 10 wt % poly(BPE)/g-C_3_N_4_ composite-modified GCE is about 110 mV, while those at the g-C_3_N_4_, 5 wt % poly(BPE)/g-C_3_N_4_, and 15 wt % poly(BPE)/g-C_3_N_4_ composite-modified GCE are 280, 247, and 155 mV, respectively (detailed data in Table 1). Meanwhile, the 10 wt % poly(BPE)/g-C_3_N_4_ composite-modified GCE presents the largest background current and peak current compared to other modified GCEs, indicating that more electrochemical active sites present on the surface of 10 wt % poly(BPE)/g-C_3_N_4_ composite-modified GCE [45]. Under the Randles–Sevcik equation, I_p_ = 2.69 × 10^5^ n^3/2^ ACD_0_^1/2^ ν^1/2^, where I_p_ is the anodic peak current, n is the number of electrons transferred, A is electroactive surface area, C is concentration, ν is the potential scanning rate, and D_0_ is the diffusion coefficient [44,46,47]. The effective surface areas of the g-C_3_N_4_, 5 wt % poly(BPE)/g-C_3_N_4_, 10 wt % poly(BPE)/g-C_3_N_4_ and 15 wt % poly(BPE)/g-C_3_N_4_ composite-modified GCE are estimated to be 0.0781 cm^2^, 0.0851 cm^2^, 0.1234 cm^2^, and 0.0989 cm^2^, respectively. The 10 wt % poly(BPE)/g-C_3_N_4_ has the largest effective surface area. Therefore, the 10 wt % poly(BPE)/g-C_3_N_4_ composite-modified GCE could be used as an excellent sensor for electroactive species.

Figure 4B shows the DPV of the differently modified GCEs in 0.1 M ABS (pH = 4.5) containing 2.0 μM Cd^2+^ and Pb^2+^. As shown, the distance between the individual peaks is large enough, with individual peaks at approximately −0.834 V and −0.586 V for Cd^2+^ and Pb^2+^, respectively. The peak current of GCE modified by 10 wt % poly(BPE)/g-C_3_N_4_ composite increased significantly, the main reason being that poly(BPE) not only effectively improves the electron transfer rate of the electrode surface, but also strongly interacts with the conjugated structure of g-C_3_N_4_ by π–π stacking, which results from electrode materials having strong adsorption capacity [48].

### 3.3. Optimization of Experimental Conditions

**Figure 5:**
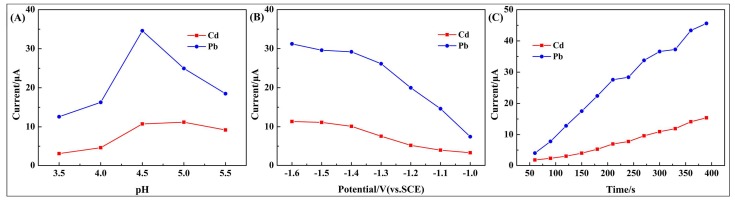
The effects of (**A**) pH values; (**B**) deposition potential; and (**C**) deposition time on the stripping peak currents of Cd^2+^ and Pb^2+^ at 10 wt % poly(BPE)/g-C_3_N_4_ composite-modified GCE in 0.1 M ABS containing 1 μM of Cd^2+^ and Pb^2+^.

To optimize the experimental conditions, simultaneous determination of 2.0 μM Cd^2+^ and Pb^2+^ at the 10 wt % poly(BPE)/g-C_3_N_4_ composite-modified GCE under different pH values were evaluated (deposition potential: −1.4 V, deposition time: 210 s, pulse width: 50 ms; pulse period: 100 ms, increment potential: 2 mV). As shown in Figure 5A, maximum current responses appeared at pH = 4.5. The lower pH values (3.5 and 4.0) could results in a reduction peak current, which is possibly due to the protonation of the hydrophilic groups reducing the absorption of metal ions. The peak current at the higher pH values (5.0 and 5.5) decreased, which is possibly due to the hydrolysis of Cd^2+^ and Pb^2+^. Thus, pH = 4.5 was chosen as the best condition for the electrochemical measurements.

The effect of the deposition potential on the performance of the modified electrode was investigated in the range from −1.0 to −1.6 V, and the results are shown in Figure 5B. The maximum current peak could be observed at −1.4 V. However, the peak currents decreased gradually with the potential moving to the negative direction. Thus, the deposition potential of −1.4 V was chosen as the optimum value for detection of two heavy metals.

Figure 5C exhibits the DPV current response of 2.0 μM Cd^2+^ and Pb^2+^ over the accumulation time of 60–390 s. As shown in Figure 5C, for the time of 60–210 s, the peak currents are almost linearly proportional to accumulation time, and this may be attributed to the fact that the amount of metal ions at the modified electrode surface greatly increases due to electrochemical deposition. When deposition time was above 210 s, the increase rate of the peak current of Pb^2+^ changed, and this is probably due to the working electrode surface saturation. Under the consideration of sensitivity, a determination time of 210 s was selected for the deposition of the ions.

### 3.4. Individual Determination of Cd^2+^ and Pb^2+^

**Figure 6:**
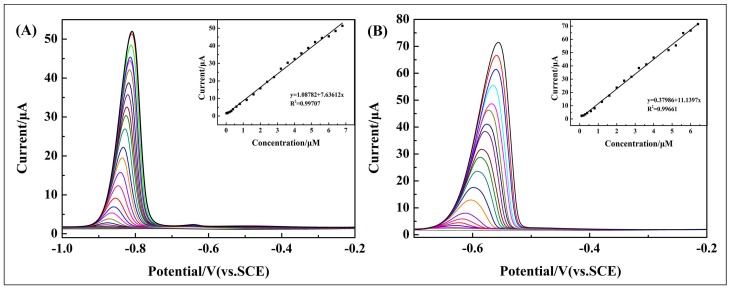
DPV response of the 10 wt % poly(BPE)/g-C_3_N_4_ composite-modified GCE for the individual analysis of (**A**) Cd^2+^ over a concentration range of 0.1–6.8 μM; (**B**) Pb^2+^ over a concentration range of 0.1–6.4 μM. The inset shows their linear equations as well as correlation coefficient.

Under the optimized conditions, DPV was used as an analytical method for the electrochemical detection of Cd^2+^ and Pb^2+^ using various modified GCEs in 0.1 M ABS (pH = 4.5). Figure 6 exhibits the DPV responses of 10 wt % poly(BEP)/g-C_3_N_4_ composite-modified GCE toward Cd^2+^ and Pb^2+^. The figure also shows the linear relationship between peak currents and concentrations of the two ions, and the inset shows as well as their linear equations and correlation coefficient (Figure 6 inset). The linear range of Cd^2+^ is 0.1–6.8 μM with a detection limit of 0.0097 μM. The linear range of Pb^2+^ is 0.1–6.4 μM with a detection limit of 0.00327 μM. Detailed results are shown in Table 2.

From the above results, it is clear that the 10 wt % poly(BPE)/g-C_3_N_4_ composite-modified electrode showed a wide detection range and low detection limit, and the detection limits were lower than the those of WHO standards. It should be noticed that the electrochemical analysis of 10 wt % poly(BPE)/g-C_3_N_4_ composite for trace metal ions may be attributed to the lone-pair electrons of nitrogen in the g-C_3_N_4_. The report shows that the highly ordered tri-s-triazine units contain many ideal coordination sites and thus metal ions can intercalate into g-C_3_N_4_ through the lone-pair electrons of nitrogen [26,27,28,29]. The combination of poly(BPE) and g-C_3_N_4_ not only improved the conduction pathway on the electrode surface, but also produced a strong conjugate effect on them, enhancing the adsorption of metal ions.

### 3.5. Simultaneous Determination of Cd^2+^ and Pb^2+^

**Figure 7:**
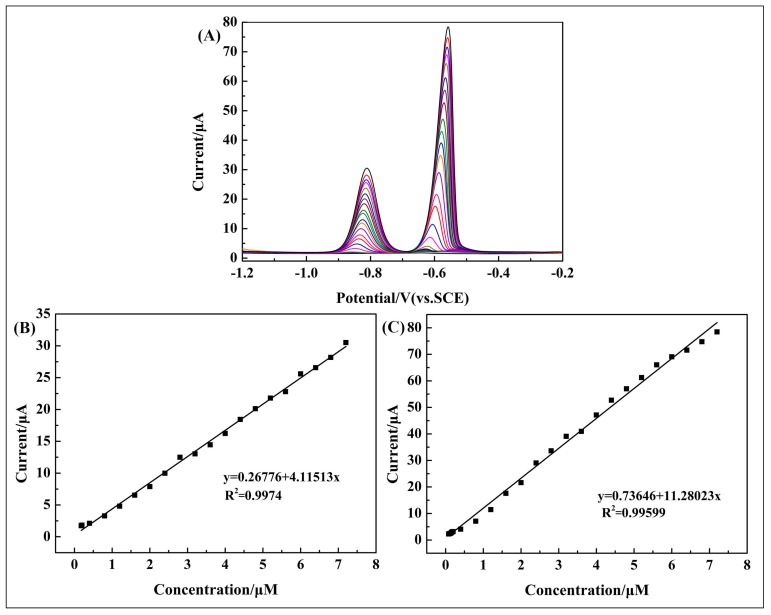
(**A**) DPV response of the 10 wt % poly(BPE)/g-C_3_N_4_ composite-modified GCE for the simultaneous analysis of Cd^2+^ and Pb^2+^ over a concentration range of 0.1–7.2 μM; (**B**,**C**) the respective calibration curves of Cd^2+^ and Pb^2+^ corresponding to (**A**).

The analytical performance of the 10 wt % poly(BPE)/g-C_3_N_4_ composite-modified GCE was investigated by simultaneous determination of Cd^2+^ and Pb^2+^ in 0.1 M ABS (pH = 4.5). As shown in Figure 7A, the current response of Cd^2+^ and Pb^2+^ appeared at −0.82 V and −0.58 V, respectively. The distance between each individual peak is large enough to simultaneous detect these heavy metal ions using the 10 wt % poly(BPE)/g-C_3_N_4_ composite-modified GCE. Figure 7B,C shows the linear relationship between peak current and concentration of the two heavy metal ions, as well as their linear equations and the correlation coefficient. The linear ranges of Cd^2+^ and Pb^2+^ are 0.12–7.2 μM and 0.08–7.2 μM, respectively. The detection limits of Cd^2+^ and Pb^2+^ are 0.018 μM and 0.00324 μM, respectively. Other detailed results are shown in Table 1.

**Figure 8:**
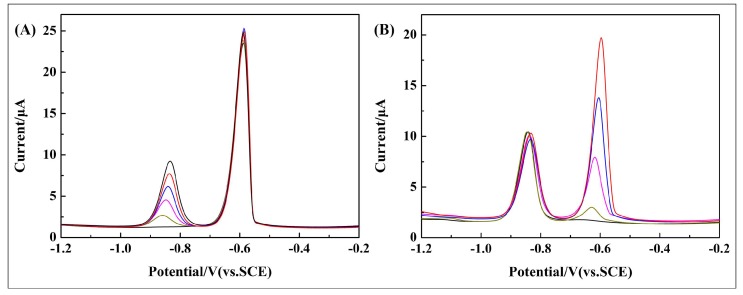
(**A**) DPV response of the 10 wt % poly(BPE)/g-C_3_N_4_ composite-modified GCE at 0, 0.4, 0.8, 1.2, 1.6, and 2 μM Cd^2+^ in the presence of 2.0 μM Pb^2+^ in 0.1 M NaAc–HAc (pH = 4.5), showing the effect of the concentration of Cd^2+^ on the peak currents of 2.0 μM Pb^2+^. (**B**) DPV response of the 10 wt % poly(BPE)/g-C_3_N_4_ composite-modified GCE at 0, 0.4, 0.8, 1.2, 1.6, and 2 μM Pb^2+^ in the presence of 2.0 μM Cd^2+^ in 0.1 M NaAc–HAc (pH = 4.5), showing the effect of the concentration of Pb^2+^ on the peak current of 2.0 μM Cd^2+^.

In the DPV technique, interfering molecules in the sample solution may be co-deposited on the active sites of the electrode surface, which result in changes in the stripping peak current. The interference may be the result of two main factors: (i) intermetallic compound formation and (ii) the competition between analytes and interferent ions for active sites on the 10 wt % poly(BPE)/g-C_3_N_4_ composite-modified GCE surface. In order to understand whether there is interference between Cd^2+^ and Pb^2+^ in simultaneous detection, we performed the following experiment. The effect of a single species on the multispecies was performed by changing one species’ concentration while the other species was unchanged. As shown in Figure 8, the DPV response of Cd^2+^ and Pb^2+^ increased linearly with the increase of the target ion’s concentration, while the other ion was kept at a constant concentration of 2 μM. From Figure 8A, it can be seen that the peak current of Pb^2+^ is practically unaltered with increasing of Cd^2+^ concentration, and the peak current of Cd^2+^ is practically unaltered with the increasing of Pb^2+^ concentration (Figure 8B). These results indicate that the other coexisting ion did not interfere with the determination of Cd^2+^ or Pb^2+^. In addition, comparisons of the differently modified electrodes toward the simultaneous detection of Cd^2+^ and Pb^2+^ are shown in Table 3. From Table 3, it can be deduced that the 10 wt % poly(BPE)/g-C_3_N_4_ composite-modified GCE could be an ideal sensor for simultaneous detection of Cd^2+^ and Pb^2+^.

### 3.6. Interference Study

It is known that, in practical applications, interference ions might co-deposit on an electrode with Cd^2+^ and Pb^2+^. The interference study was performed by adding various potentially interfering metal cations including Na^+^, K^+^, Ca^2+^, Mg^2+^, Al^3+^, Fe^3+^, Co^2+^, Ni^2+^, Cu^2+^, and Zn^2+^ in 50-fold excess with target metal ions into a standard solution containing 2 μM Cd^2+^ and Pb^2+^ under the optimized working conditions. As listed in Table 4, the change in the peak current of Cd^2+^ and Pb^2+^ was less than 10% after adding interfering ions. Thus, the 10 wt % poly(BPE)/g-C_3_N_4_ composite-modified GCE displayed high selectivity for Cd^2+^ and Pb^2+^ in the heavy metal analysis.

### 3.7. Reproducibility of Modified Electrode

**Figure 9:**
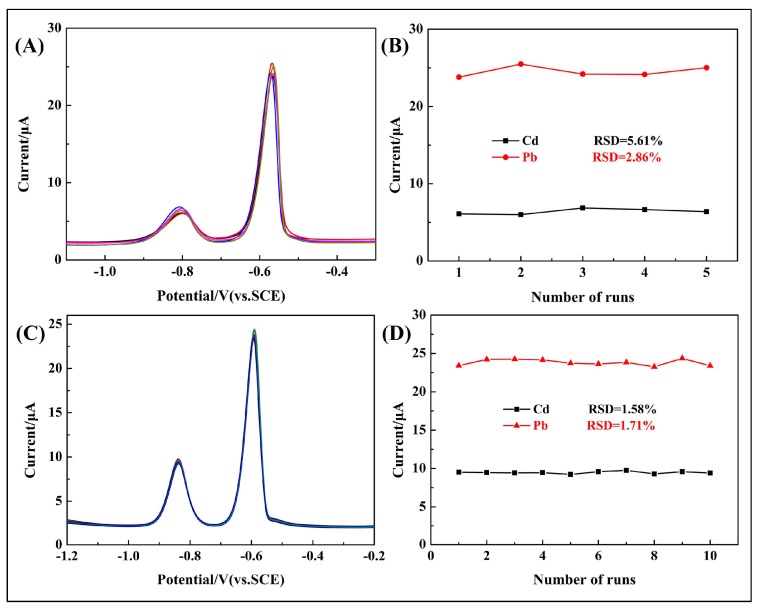
(**A**) DPV response of 2.0 μM Cd^2+^ and Pb^2+^ on five 10 wt % poly(BPE)/g-C_3_N_4_ composite-modified GCEs in 0.1 M ABS (pH = 4.5). (**B**) The data collected from every DPV response for a total of 10 times. RSD refers to the relative standard deviation. (**C**) DPV response of 2.0 μM Cd^2+^ and Pb^2+^ on the 10 wt % poly(BPE)/g-C_3_N_4_ composite-modified GCE in 0.1 M ABS (pH = 4.5). (**D**) The data collected from every DPV response for a total of 10 times, RSD refers to the relative standard deviation.

To further evaluate the sensing performance, the repeatability of the 10 wt % poly(BPE)/g-C_3_N_4_ composite-modified GCE was tested with 2 μM Cd^2+^ and Pb^2+^ under the optimized conditions. Electrolyte: 0.1 M ABS (pH = 4.5), deposition potential: −1.4 V, deposition time: 210 s. As shown in Figure 9, the reproducibility was estimated with five different 10 wt % poly(BPE)/g-C_3_N_4_ composite-modified GCEs that were prepared independently by the same procedure. The values of relative standard deviation (RSD) were 5.61% for Cd^2+^ and 2.86% for Pb^2+^ in the presence of 2 μM of metal ions, which demonstrated the reliability of the fabrication procedure. Repeatability of the developed method was evaluated by detecting 2 μM Cd^2+^ and Pb^2+^ at the 10 wt % poly(BPE)/g-C_3_N_4_ composite-modified GCE for 10 measurements. The values of RSD were 1.58% for Cd^2+^ and 1.71% for Pb^2+^. Hence, the 10 wt % poly(BPE)/g-C_3_N_4_ composite-modified GCE shows ideal reproducibility and repeatability.

### 3.8. Real Sample Analysis

The 10 wt % poly(BPE)/g-C_3_N_4_ composite-modified GCE for simultaneous determination of Cd^2+^ and Pb^2+^ showed high sensitivity and better reproducibility. Tap water samples were taken to carry out a further study. Firstly, certain amounts of tap water in 0.1 M ABS (pH = 4.5) were prepared. Subsequently, standard solutions of Cd^2+^ and Pb^2+^ with different concentration were added to the tap water samples. The results are illustrated in Table 5. The recoveries of the Cd^2+^ and Pb^2+^ are 98.64–106.74% and 99.81–113.15%, respectively. Results indicate that the 10 wt % poly(BPE)/g-C_3_N_4_ composite-modified GCE could be applied in the detection of Cd^2+^ and Pb^2+^ in tap water samples.

## 4. Conclusions

In summary, a novel poly(BPE)/g-C_3_N_4_ composite has been successfully synthesized via chemical oxidative polymerization and used for determination of Cd^2+^ and Pb^2+^. The highly ordered tri-s-triazine units contain many ideal coordination sites, and thus metal ions can intercalate into g-C_3_N_4_ through the lone-pair electrons of nitrogen. Besides that, the composite-modified electrode possesses a wide detection range and excellent sensitivity towards the simultaneous detection of Cd^2+^ and Pb^2+^.The results demonstrate that the 10 wt % poly(BPE)/g-C_3_N_4_ composite-modified electrode possesses high sensitively, wide linear range, and low detection limit for the determination of Cd^2+^ and Pb^2+^.

## Figures and Tables

**Table 1:** The peak-to-peak potential (ΔE_p_) of differently modified GCEs.

Modified GCE	E_anodicpeak_ (mV)	E_cathodicpeak_ (mV)	∆E_p_ (mV)
g-C_3_N_4_	305	225	280
5 wt % poly(BPE)/g-C_3_N_4_	295	48	247
10 wt % poly(BPE)/g-C_3_N_4_	220	110	110
15 wt % poly(BPE)/g-C_3_N_4_	249	94	155

**Table 2:** Performance of the 10 wt % poly(BPE)/g-C_3_N_4_ composite-modified GCE for individual and simultaneous determination of Cd^2+^ and Pb^2+^.

Types	Analytes	Linear Range (μM)	Linear Regression Equation	R^2^	Detection Limit (μM)
Individual determination	Cd^2+^	0.1–6.8	I (μA) = 1.08782 + 7.63612c (μM)	0.99707	0.00970
	Pb^2+^	0.1–6.4	I (μA) = 0.37986 + 11.1397c (μM)	0.99661	0.00327
Simultaneous determination	Cd^2+^	0.12–7.2	I (μA) = 0.26776 + 4.11513c (μM)	0.99740	0.0180
	Pb^2+^	0.08–7.2	I (μA) = 0.73646 + 11.28023c (μM)	0.99599	0.00324

**Table 3:** Comparison of different sensors for the determination of heavy metals.

Electrode	Methods	Analytes	Linear Range (μM)	LOD (μM)	Ref.
PA/PPy/GO	DPV	Cd^2+^	0.045–1.335	0.019	[43]
		Pb^2+^	0.024–0.724	0.002	
l-cys/GR-CS/GCE	DPASV	Cd^2+^	0.005–0.6	0.0012	[4]
		Pb^2+^	0.005–0.3	0.0072	
GO/ex-Bi_2_Te_3_ modified GCEs	SWV	Cd^2+^	0.009–0.178	0.0018	[49]
		Pb^2+^	0.0024–0.0965	0.00048	
G/PANI/PS fiber/SPCE	SWASV	Cd^2+^	0.089–4.448	0.039	[50]
		Pb^2+^	0.048–2.413	0.016	
AuNPs/CNFs	SWASV	Cd^2+^	0.1–1.0	0.1	[51]
		Pb^2+^	0.1–1.0	0.1	
DCD-CPE	DPSV	Cd^2+^	0.25–25	0.04	[52]
		Pb^2+^	0.1–15	0.01	
Nafion-HAP	DPASV	Cd^2+^	0.1–10.0	0.035	[53]
		Pb^2+^	0.1–10.0	0.049	
BT-SBA-15/CPE	DPASV	Cd^2+^	2.0–10.0	0.4	[54]
		Pb^2+^	0.3–7.0	0.04	
Poly(BPE)/g-C_3_N_4_	DPV	Cd^2+^	0.12–7.2	0.018	This work
		Pb^2+^	0.08–7.2	0.00324	

PA/PPy/GO: phytic acid-functionalized polypyrrole/graphene oxide-modified electrode; l-cys/GR-CS/GCE: l-cysteine/graphene–chitosan-modified GCE; GO/ex-Bi_2_Te_3_-modified GCE: GO/exfoliated Bi_2_Te_3_-modified GCE; G/PANI/PS nanoporous fiber/SPCE: graphene/polyaniline/polystyrene nanoporous fiber-modified screen-printed carbon electrode; AuNPs/CNFs: a well-dispersed Au nanoparticle grown on carbon nanofibers; BT-SBA-15/CPE: 2-benzothiazolethiol functionalized SBA-15; DCD-CPE: diacetyldioxime-modified carbon paste electrode; HAP-Nafion: the nanocomposite of HAP and Nafion.

**Table 4:** Interferences of some metal ions (100 μM) on the 10 wt % poly(BPE)/g-C_3_N_4_ composite-modified GCE during simultaneous determination of 2 μM Cd^2+^ and Pb^2+^. Electrolyte: 0.1 M ABS (pH = 4.5); deposition potential: −1.4 V; deposition time: 210 s.

Interferences	Contribution (%) (Ip Cd^2+^ = 100%)	Contribution (%) (Ip Pb^2+^ = 100%)
Na	+3.15%	+9.46%
K	−8.77%	+2.85%
Ca	−8.81%	+3.92%
Mg	+6.11%	−2.64%
Al	−8.36%	1.31%
Fe	5.32%	0.5%
Co	−0.75%	−0.7%
Ni	4.94%	8.8%
Cu	−13.86%	−5.38%
Zn	−4.19%	5.9%

**Table 5:** Determination of Cd^2+^ and Pb^2+^ in tap water.

Original	Added (μM)		Found (μM)		Recovery (%)	
(μM)	Cd^2+^	Pb^2+^	Cd^2+^	Pb^2+^	Cd^2+^	Pb^2+^
N.D	1	1	1.0396	1.1315	103.96	113.15
N.D	2	2	2.1347	2.0334	106.74	101.67
N.D	3	3	3.0925	2.9944	103.08	99.81
N.D	4	4	3.9455	4.0102	98.64	100.26

N.D: not detected.

## References

[1] Shahbazi Y., Ahmadi F., Fakhari F. (2016). Voltammetric determination of Pb, Cd, Zn, Cu and Se in milk and dairy products collected from Iran: An emphasis on permissible limits and risk assessment of exposure to heavy metals. Food Chem..

[2] Khan I., Pandit U.J., Wankar S., Limaye S.N. (2017). Centrifugation assisted digestion for simultaneous voltammetric determination of ultra trace metal ions in water and milk samples. Environ. Nanotechnol. Monit. Manag..

[3] Mahesar S.A., Sherazi S.T., Niaz A., Bhanger M.I., Uddin S., Rauf A. (2010). Simultaneous assessment of Zinc, Cadmium, Lead and Copper in poultry feeds by differential pulse anodic stripping voltammetry. Food Chem. Toxicol..

[4] Guo Z., Li D.D., Luo X.K., Li Y.H., Zhao Q.N., Li M.M., Zhao Y.T., Sun T.S., Ma C. (2016). Simultaneous determination of trace Cd(II), Pb(II) and Cu(II) by differential pulse anodic stripping voltammetry using a reduced graphene oxide-chitosan/poly-l-lysine nanocomposite modified glassy carbon electrode. J. Colloid Interface Sci..

[5] Lu Y., Liang X., Niyungeko C., Zhou J., Tian G. (2017). A review of the identification and detection of heavy metal ions in the environment by voltammetry. Talanta.

[6] Koelmel J., Amarasiriwardena D. (2012). Imaging of metal bioaccumulation in hay-scented fern (*Dennstaedtia punctilobula*) rhizomes growing on contaminated soils by laser ablation ICP-MS. Environ. Pollut..

[7] Massadeh A.M., Alomary A.A., Mir S., Momani F.A., Haddad H.I., Hadad Y.A. (2016). Analysis of Zn, Cd, As, Cu, Pb, and Fe in snails as bioindicators and soil samples near traffic road by ICP-OES. Environ. Sci. Poll. Res..

[8] Sánchezrodas D., Corns W.T., Chen B., Stockwell P.B. (2010). Atomic Fluorescence Spectrometry: A suitable detection technique in speciation studies for arsenic, selenium, antimony and mercury. J. Anal. At. Spectrom..

[9] Siraj K., Kitte S.A. (2013). Analysis of Copper, Zinc and Lead using Atomic Absorption Spectrophotometer in ground water of Jimma town of Southwestern Ethiopia. Int. J. Chem. Anal. Sci..

[10] Grasso G., D’Urso L., Messina E., Cataldo F. (2009). A mass spectrometry and surface enhanced Raman spectroscopy study of the interaction between linear carbon chains and noble metals. Carbon.

[11] Caschera D., Federici F., Zane D., Focanti F., Curulli A., Padeletti G. (2008). Gold nanoparticles modified GC electrodes: Electrochemical behaviour dependence of different neurotransmitters and molecules of biological interest on the particles size and shape. J. Nanopart. Res..

[12] Wei Y., Yang R., Yu X.Y., Wang L., Liu J.H., Huang X.J. (2012). Stripping voltammetry study of ultra-trace toxic metal ions on highly selectively adsorptive porous magnesium oxide nanoflowers. Analyst.

[13] Zhao D., Guo X., Wang T., Alvarez N., Shanov V.N., Heineman W.R. (2014). Simultaneous Detection of Heavy Metals by Anodic Stripping Voltammetry Using Carbon Nanotube Thread. Electroanalysis.

[14] Gadgil B., Damlin P., Dmitrieva E., Ääritalo T., Kvarnström C. (2016). Exploring amide linkage in a polyviologen derivative towards simultaneous voltammetric determination of Pb(II), Cu(II) and Hg(II) ions. Electrochim. Acta.

[15] Xie Y.L., Zhao S.Q., Ye H.L., Yuan J., Song P., Hu S.Q. (2015). Graphene/CeO_2_ hybrid materials for the simultaneous electrochemical detection of Cadmium(II), Lead(II), Copper(II), and Mercury(II). J. Electroanal. Chem..

[16] Ruecha N., Rodthongkum N., Cate D.M., Volckens J., Chailapakul O., Henry C.S. (2015). Sensitive electrochemical sensor using a graphene–polyaniline nanocomposite for simultaneous detection of Zn(II), Cd(II), and Pb(II). Anal. Chim. Acta.

[17] Sun C., Zhang M., Fei Q., Wang D., Sun Z., Geng Z., Xu W., Liu F. (2017). Graphite-like g-C_3_N_4_-F127-Au Nanosheets Used for Sensitive Monitoring of Heat Shock Protein 90. Sens. Actuators B Chem..

[18] Zheng Y., Zhang Z., Li C. (2017). A comparison of graphitic carbon nitrides synthesized from different precursors through pyrolysis. J. Photochem. Photobiol. Chem..

[19] Xu L., Xia J., Wang L., Ji H., Qian J., Xu H., Wang K., Li H. (2014). Graphitic Carbon Nitride Nanorods for Photoelectrochemical Sensing of Trace Copper(II) Ions. Eur. J. Inorg. Chem..

[20] Wu M., Wang Q., Sun Q., Jena P. (2013). Functionalized Graphitic Carbon Nitride for Efficient Energy Storage. J. Phys. Chem. C.

[21] Wang X., Maeda K., Thomas A., Takanabe K., Xin G., Carlsson J.M., Domen K., Antonietti M. (2009). A metal-free polymeric photocatalyst for hydrogen production from water under visible light. Nat. Mater..

[22] Liu Q., Zhang J. (2013). Graphene Supported Co-g-C_3_N_4_ as a Novel Metal–Macrocyclic Electrocatalyst for the Oxygen Reduction Reaction in Fuel Cells. Langmuir.

[23] Shalom M., Inal S., Fettkenhauer C., Neher D., Antonietti M. (2013). Improving carbon nitride photocatalysis by supramolecular preorganization of monomers. J. Am. Chem. Soc..

[24] Feng Q.M., Shen Y.Z., Li M.X., Zhang Z.L., Zhao W., Xu J.J., Chen H.Y. (2016). Dual-Wavelength Electrochemiluminescence Ratiometry Based on Resonance Energy Transfer between Au Nanoparticles Functionalized g-C_3_N_4_ Nanosheet and Ru(bpy)_3_^2+^ for microRNA Detection. Anal. Chem..

[25] Tian J., Liu Q., Asiri A.M., Alyoubi A.O., Sun X. (2013). Ultrathin graphitic carbon nitride nanosheet: A highly efficient fluorosensor for rapid, ultrasensitive detection of Cu^2+^. Anal. Chem..

[26] Gao W., Wang X., Li P., Wu Q., Wu S., Yu Y., Ding K. (2016). Highly sensitive and selective detection of cadmium with a graphite carbon nitride nanosheets/Nafion electrode. RSC Adv..

[27] Wang D.P., Tang Y., Zhang W.D. (2013). A carbon nitride electrode for highly selective and sensitive determination of lead(II). Microchim. Acta.

[28] Amiri M., Salehniya H., Habibiyangjeh A. (2016). Graphitic Carbon Nitride/Chitosan Composite for Adsorption and Electrochemical Determination of Mercury in Real Samples. Ind. Eng. Chem. Res..

[29] Lu X., Yang Y., Liu T. (2011). Synthesis, Al/Mg Intercalation and Structure Study of Graphite-like Carbon Nitride. J. Mater. Sci. Technol..

[30] Sun Y., Jiang J., Liu Y., Wu S., Zou J. (2017). A facile one-pot preparation of Co_3_O_4_/g-C_3_N_4_ heterojunctions with excellent electrocatalytic activity for the detection of environmental phenolic hormones. Appl. Surf. Sci..

[31] Zhang H., Huang Q., Huang Y., Li F., Zhang W., Wei C., Chen J., Dai P., Huang L., Huang Z. (2014). Graphitic carbon nitride nanosheets doped graphene oxide for electrochemical simultaneous determination of ascorbic acid, dopamine and uric acid. Electrochim. Acta.

[32] Chidhambaram N., Ravichandran K. (2017). Single step transformation of urea into metal-free g-C_3_N_4_ nanoflakes for visible light photocatalytic applications. Mater. Lett..

[33] Dubois C.J., Reynolds J.R. (2010). 3,4-Ethylenedioxythiophene–Pyridine-Based Polymers: Redox or n-Type Electronic Conductivity?. Adv. Mater..

[34] Boydis M.J., Müller J.O., Antonietti M., Arne T. (2008). Ionothermal synthesis of crystalline, condensed, graphitic carbon nitride. Chemistry.

[35] Martha S., Nashim A., Parida K.M. (2013). Facile synthesis of highly active g-C_3_N_4_ for efficient hydrogen production under visible light. J. Mater. Chem. A.

[36] Zhou X., Jin B., Li L., Peng F., Wang H., Yu H., Fang Y. (2012). A carbon nitride/TiO_2_ nanotube array heterojunction visible-light photocatalyst: Synthesis, characterization, and photoelectrochemical properties. J. Mater. Chem..

[37] Liu F., Jamal R., Wang Y., Wang M., Yang L., Abdiryim T. (2016). Photodegradation of methylene blue by photocatalyst of D-A-D type polymer/functionalized multi-walled carbon nanotubes composite under visible-light irradiation. Chemosphere.

[38] Wang Y., Jamal R., Wang M., Yang L., Liu F., Abdiryim T. (2017). A donor–acceptor–donor-type conjugated polymer-modified TiO_2_ with enhanced photocatalytic activity under simulated sunlight and natural sunlight. J. Mater. Sci..

[39] Yang L., Jamal R., Liu F., Wang Y., Abdiryim T. (2017). Structure and photocatalytic activity of a low band gap donor–acceptor–donor (D–A–D) type conjugated polymer: Poly(EDOT–pyridazine–EDOT). RSC Adv..

[40] Xu J., Chen Y., Ma D., Shang J.K., Li Y.X. (2017). Simple preparation of MgO/g-C_3_N_4_ catalyst and its application for catalytic synthesis of dimethyl carbonate via transesterification. Catal. Commun..

[41] Dong Q., Chen Y., Wang L., Ai S., Ding H., Dong Q., Chen Y., Wang L., Ai S., Ding H. (2017). Cu-modified alkalinized g-C_3_N_4_ as photocatalytically assisted heterogeneous Fenton-like catalyst. Appl. Surf. Sci..

[42] Wang L., Ma C., Guo Z., Lv Y., Chen W., Chang Z., Yuan Q., Ming H., Wang J. (2017). In-situ growth of g-C_3_N_4_ layer on ZnO nanoparticles with enhanced photocatalytic performances under visible light irradiation. Mater. Lett..

[43] Dai H., Wang N., Wang D., Ma H., Lin M. (2016). An electrochemical sensor based on phytic acid functionalized polypyrrole/graphene oxide nanocomposites for simultaneous determination of Cd(II) and Pb(II). Chem. Eng. J..

[44] Wang C., Du J., Wang H., Zou C.E., Jiang F., Yang P., Du Y. (2014). A facile electrochemical sensor based on reduced graphene oxide and Au nanoplates modified glassy carbon electrode for simultaneous detection of ascorbic acid, dopamine and uric acid. Sens. Actuators B Chem..

[45] Ali A., Jamal R., Abdiryim T., Huang X. (2017). Synthesis of monodispersed PEDOT/Au hollow nanospheres and its application for electrochemical determination of dopamine and uric acid. J. Electroanal. Chem..

[46] Xu F., Fan W., Yang D., Yong G., Li H. (2014). Electrochemical sensing platform for L-CySH based on nearly uniform Au nanoparticles decorated graphene nanosheets. Mater. Sci. Eng. C.

[47] Wang L., Yamauchi Y. (2009). Facile Synthesis of Three-Dimensional Dendritic Platinum Nanoelectrocatalyst. Chem. Mater..

[48] Zhao G., Yin Y., Wang H., Liu G., Wang Z. (2016). Sensitive stripping voltammetric determination of Cd(II) and Pb(II) by a Bi/multi-walled carbon nanotube-emeraldine base polyaniline-Nafion composite modified glassy carbon electrode. Electrochim. Acta.

[49] Tseliou F., Avgeropoulos A., Falaras P., Prodromidis M.I. (2017). Low dimensional Bi_2_Te_3_-graphene oxide hybrid film-modified electrodes for ultra-sensitive stripping voltammetric detection of Pb(II) and Cd(II). Electrochim. Acta.

[50] Promphet N., Rattanarat P., Rangkupan R., Chailapakul O., Rodthongkum N. (2015). An electrochemical sensor based on graphene/polyaniline/polystyrene nanoporous fibers modified electrode for simultaneous determination of lead and cadmium. Sens. Actuators B Chem..

[51] Zhang B., Chen J.D., Zhu H., Yang T.T., Zou M.L., Zhang M., Du M.L. (2016). Facile and green fabrication of size-controlled AuNPs/CNFs hybrids for the highly sensitive simultaneous detection of heavy metal ions. Electrochim. Acta.

[52] Hu C., Wu K., Dai X., Hu S. (2003). Simultaneous determination of lead(II) and cadmium(II) at a diacetyldioxime modified carbon paste electrode by differential pulse stripping voltammetry. Talanta.

[53] Gao F., Gao N., Nishitani A., Tanaka H. (2016). Rod-like hydroxyapatite and Nafion nanocomposite as an electrochemical matrix for simultaneous and sensitive detection of Hg^2+^, Cu^2+^, Pb^2+^ and Cd^2+^. J. Electroanal. Chem..

[54] Cesarino I., Marino G., Matos J.R., Cavalheiro E.T. (2008). Evaluation of a carbon paste electrode modified with organofunctionalised SBA-15 nanostructured silica in the simultaneous determination of divalent lead, copper and mercury ions. Talanta.

